# Cardiac and skeletal muscle manifestations in the G608G mouse model of Hutchinson‐Gilford progeria syndrome

**DOI:** 10.1111/acel.14259

**Published:** 2024-07-03

**Authors:** Yeojin Hong, Alice Rannou, Nancy Manriquez, Jack Antich, Weixin Liu, Mario Fournier, Ariel Omidfar, Russell G. Rogers

**Affiliations:** ^1^ Smidt Heart Institute Cedars‐Sinai Medical Center Los Angeles California USA

**Keywords:** cardiomyopathy, fibroblasts, G608G HGPS mouse model, Hutchinson‐Gilford progeria syndrome, myopathy, senescence

## Abstract

Hutchinson‐Gilford progeria syndrome (HGPS) is a rare premature aging disorder resulting from de novo mutations in the lamin A gene. Children with HGPS typically pass away in their teenage years due to cardiovascular diseases such as atherosclerosis, myocardial infarction, heart failure, and stroke. In this study, we characterized the G608G HGPS mouse model and explored cardiac and skeletal muscle function, along with senescence‐associated phenotypes in fibroblasts. Homozygous G608G HGPS mice exhibited cardiac dysfunction, including decreased cardiac output and stroke volume, and impaired left ventricle relaxation. Additionally, skeletal muscle exhibited decreased isometric tetanic torque, muscle atrophy, and increased fibrosis. HGPS fibroblasts showed nuclear abnormalities, decreased proliferation, and increased expression of senescence markers. These findings provide insights into the pathophysiology of the G608G HGPS mouse model and inform potential therapeutic strategies for HGPS.

## Graphical abstract

Homozygous G608G HGPS mice demonstrated cardiac abnormalities such as reduced cardiac output and stroke volume, and compromised left ventricle relaxation. Their skeletal muscles showed reduced isometric tetanic torque, muscle atrophy, and fibrosis. Furthermore, HGPS fibroblasts exhibited nuclear irregularities, decreased cell proliferation, and higher levels of senescence markers. These observations shed light on the disease mechanisms in the G608G HGPS mouse model and guide potential therapeutic approaches for HGPS.

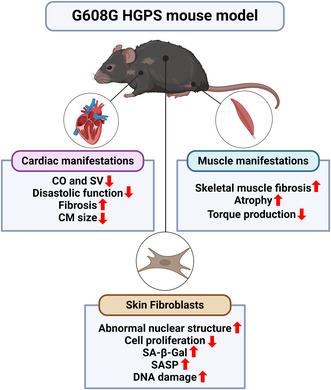

**Abbreviations**
CM: cardiomyocyte
CO: cardiac output
DNA: deoxyribonucleic acid
HGPS: Hutchinson‐Gilford progeria syndrome
SASP: senescence associated secretory phenotype
SA‐β‐Gal: senescence associated β‐galactosidase
SV: stroke volume

## 1 INTRODUCTION

Hutchinson‐Gilford progeria syndrome (HGPS) is a rare autosomal dominant premature aging disorder caused by de novo mutations in the lamin A gene (LMNA) (most commonly c.1824C > T, p.G608G) (Eriksson et al., 2003; Goldman et al., 2004). Point mutation of LMNA, c.1824C > T, p.G608G, induces aberrant splicing of exon 11, causing a 50 amino acid deletion and incorrect post‐translational processing (Eriksson et al., 2003). As a result, a farnesylated truncated protein called progerin accumulates in the nuclear envelope, causing abnormalities in nuclear morphology. Cellular defects such as increased DNA damage, loss of chromatin structure, telomere shortening, activation of senescence pathways, and alterations of metabolic pathways are hallmarks of HGPS (Arancio et al., 2014; Decker et al., 2009; Kreienkamp & Gonzalo, 2020; Kubben & Misteli, 2017; Liu et al., 2008).

Clinical presentations in HGPS patients include premature cardiac disease (our focus here), alopecia, scleroderma, swollen and stiff joints, muscle weakness, pigmented spots, and lipodystrophy (Kreienkamp & Gonzalo, 2020). Children diagnosed with HGPS typically succumb to cardiovascular diseases (CVD) in their teenage years, including atherosclerosis, myocardial infarction, heart failure, and stroke (Gordon et al., 2014). CVD in HGPS patients develops as a result of the loss of vascular smooth muscle cells (VSMCs), endothelial cell dysfunction, cardiovascular calcification, perivascular fibrosis and stiffening, and cardiac electrical abnormalities (Hanumanthappa et al., 2011; Merideth et al., 2008; Olive et al., 2010; Rivera‐Torres et al., 2016; Song et al., 2014; Stehbens et al., 1999; Stehbens et al., 2001).

Due to the rarity of HGPS, conducting research and clinical trials is challenging; however, utilizing animal models of HGPS becomes essential for developing therapeutic agents. Humanized HGPS mice were created by transducing B6 mice with a 164.4 kilobase bacterial artificial chromosome harboring the most common human LMNA mutation (c.1824C > T, p.G608G) (Varga et al., 2006). These mice simultaneously produce both human progerin and mouse lamin A/C, and thus, genocopy human HGPS. Homozygotes (LMNA^G/G^) with two copies of the human LMNA G608G showed severe growth defects, lower survivability, decreased dermal and subcutaneous adipose tissue, and loss of VSMCs compared to hemizygotes (LMNA^G/+^) and wild‐type (LMNA^+/+^) mice (Cabral et al., 2021). However, it is currently unknown if these mice also develop cardiac and skeletal muscle pathology. In this study, we sought to characterize cardiac and skeletal muscle function and structure in humanized HGPS mice. The discoveries reported here provide further support of G608G HGPS mice to model hallmark features of clinical HGPS.

## 2 MATERIALS AND METHODS

### 2.1 Mice

Hemizygote (*LMNA* ^G/+^) HGPS mice carrying a bacterial artificial chromosome containing the human G608G (c.1824C > T) LMNA gene mutation were purchased from The Jackson Laboratory (#010667). Hemizygotes were bred to generate homozygotes (*LMNA* ^G/G^; hereafter referred to as HGPS) and wild‐type (*LMNA* ^+/+^) littermates. To incorporate sex as a biological variable, both male and female mice were used in this study. All animal procedures were approved by Cedars‐Sinai Medical Center's Institutional Animal Care and Use Committee.

### 2.2 Cells

Mouse primary fibroblasts were isolated and cultured, as previously described (Seluanov et al., 2010). Briefly, after euthanizing mice, underarm skin was collected and tissue fragments were cut using a sterile scalpel. Tissue fragments were then incubated with 0.14 U/mL Liberase™ TL (Roche) for 1 h at 37°C in a rotating water bath. After incubation, the enzyme reaction was stopped using complete media (Dulbecco's Modified Eagle Medium [DMEM, Thermo Fisher Scientific] containing 10% fetal bovine serum [HyClone] and 1X penicillin/streptomycin [Thermo Fisher Scientific]). Digested tissue fragments were centrifuged at 500*g* for 5 min. Tissue pellets were resuspended in complete media and transferred to a 150 mm tissue culture dish and incubated in a humidified atmosphere of 5% CO_2_ at 37°C. Human HGPS fibroblasts (#AG06917) were obtained from a 3‐year‐old male donor with a de novo heterozygous silent mutation Gly608Gly (G608G) in exon 11 of LMNA. Unaffected human fibroblasts (#AG06299) were obtained from the 34‐year‐old mother of the proband AG06917. All human primary fibroblasts were purchased from Coriell Institute, and were cultured in complete media in a humidified incubator with 5% CO_2_ at 37°C. For TGF‐β1 activation, mouse primary fibroblasts were plated and incubated for 24 h in serum‐free DMEM. Subsequently, the media were replaced with DMEM supplemented with 2% FBS and then cells were incubated with PBS or 10 ng/mL mouse recombinant TGF‐β1 (BioLegend) for 48 h, then total RNA was extracted using the miRNeasy Mini Kit (Qiagen), according to the manufacturer's protocol.

### 2.3 Echocardiography

Heart function was measured by transthoracic echocardiography (Vevo 3100, Visual Sonics) at 8 weeks of age and then every 4 weeks until the study endpoint. Systolic function was measured in M‐mode along the long‐axis. Diastolic function was measured by pulse‐wave doppler (E and A wave) and tissue doppler mode (E' wave) (Cho et al., 2017).

### 2.4 Muscle function

We evaluated contractile function of the left anterior crural muscles, including the tibialis anterior (TA), extensor digitorum longus, and extensor hallucis muscles, as described (Baumann et al., 2014; Rogers et al., 2015). Briefly, mice were anesthetized with isoflurane (1.5% isoflurane and 400 mL O_2_/min) and positioned on a temperature‐controlled platform maintaining core body temperature at 37°C. The left knee was clamped, and the left foot was secured to an aluminum “shoe” connected to the shaft of an Aurora Scientific 300C servomotor (Aurora Scientific). Sterilized needles were inserted through the skin to stimulate the left common peroneal nerve. Stimulation voltage and needle electrode placement were optimized with 5–15 isometric contractions (200 ms train of 0.1 ms pulses at 300 Hz). Following optimization, contractile function of the anterior crural muscles was assessed by measuring isometric torque as a function of stimulation frequency (20–300 Hz), as described (Baumann et al., 2016).

### 2.5 Masson's trichrome staining

The heart and TA were dissected from 28‐week‐old mice and tissues were embedded in Tissue‐Tek® O.C.T, and frozen with 2‐methylbutane precooled in liquid nitrogen, as described (Rogers & Otis, 2017). Heart and TA cryoblocks were cut using a cryostat (CM3050S, Leica). Sections were post‐fixed with 10% neutral‐buffered formalin for 30 min and staining was performed using Weigert's Iron Hematoxylin Set (HT1079, Sigma‐Aldrich) and Trichrome Stain (Masson) Kit (HT15, Sigma‐Aldrich), according to the manufacturer's protocol.

### 2.6 Hydroxyproline assay

The heart and TA were dissected from 28‐week‐old mice and tissues were frozen with liquid nitrogen and stored at −80°C. Hydroxyproline was measured on frozen tissues using a Hydroxyproline Assay Kit (#ab222941, Abcam), according to the manufacturer's protocol.

### 2.7 Immunohistochemistry

Heart and TA cryosections from 28‐week‐old mice were used for immunohistochemistry. Sections were post‐fixed with 10% neutral‐buffered formalin for 30 min and permeabilized with 0.01% Triton X‐100. Specimens were then incubated with protein block (#ab64226, Abcam) for 1 h, followed by primary antibodies: anti‐collagen I antibody (#ab21286, Abcam) and anti‐collagen III antibody (#PA5‐34787, Thermo Fisher Scientific), overnight at 4°C. Then, specimens were incubated with an Alexa Fluor 488‐conjugated secondary antibody (#A21206, Thermo Fisher Scientific) for 2 h at room temperature and mounted with Fluoroshield plus DAPI (Sigma‐Aldrich). Images were captured using a multi‐photon confocal fluorescence microscope (Leica) and quantitated using ImageJ software (National Institutes of Health).

### 2.8 Senescence‐associated β‐galactosidase (SA‐β‐gal) staining

Human and mouse fibroblasts were seeded in a 12‐well cell culture plate containing 0.5 mL of complete media. Then, cells were stained using the Senescence β‐Galactosidase Staining Kit (#9860, Cell Signaling Technology), according to the manufacturer's protocol. Cell images were captured using a Cytation 5 (BioTek).

### 2.9 Immunocytochemistry

Fibroblasts were plated in Falcon 4‐well Culture Slide (Corning) and incubated overnight in a humidified atmosphere of 5% CO_2_ at 37°C. Then, cells were fixed with 4% paraformaldehyde in PBS (pH 7.4) for 10 min and permeabilized with 0.01% Triton X‐100. Cells were then incubated with protein block (#ab64226, Abcam) for 1 h, followed by primary antibodies lamin A/C antibody for mouse (#sc‐6215, Santa Cruz), lamin A/C antibody for human and homozygous mouse (#MA3‐1000, Thermo Fisher Scientific), p16INK4a antibody (#ab211542, Abcam), p21 antibody (#ab188224, Abcam), p‐γH2AX antibody (#9718, Cell Signaling Technology), overnight at 4°C. Then, cells were incubated with Alexa Fluor 488‐conjugated secondary antibodies for 2 h at room temperature and mounted with Fluoroshield plus DAPI (Sigma‐Aldrich). Cell images were captured using a multi‐photon confocal fluorescence microscope (Leica) and quantitated using ImageJ software (National Institutes of Health).

### 2.10 EdU cell proliferation assay

Cell proliferation was measured using the Click‐iT EdU Cell Proliferation Kit (Thermo Fisher Scientific), according to the manufacturer's protocol. Briefly, human and mouse fibroblasts were seeded in a Falcon 4‐well Culture Slide (Corning) and incubated with 10 μM of EdU solution for 4 h in a humidified atmosphere of 5% CO_2_ at 37°C. After fixation and permeabilization, cells were mounted with Fluoroshield plus DAPI (Sigma‐Aldrich). Cell images were captured using a multi‐photon confocal fluorescence microscope (Leica) and quantitated using ImageJ software (National Institutes of Health).

### 2.11 Western blotting

Fibroblasts were washed with PBS and protein was extracted using RIPA Lysis and Extraction Buffer (Thermo Fisher Scientific) with Halt Protease and Phosphatase Inhibitor Cocktail (Thermo Fisher Scientific), according to the manufacturer's protocol. Protein concentration of the cell lysates was measured using the Pierce BCA protein assay kit (Thermo Fisher Scientific). Protein lysates (20 μg) were separated, as previously described (Rogers et al., 2019), by polyacrylamide gel electrophoresis (4%–12%) and transferred to polyvinylidene difluoride membranes. Blots were blocked with 5% non‐fat dried milk (Bio‐Rad) in TBS‐T (Thermo Fisher Scientific) and incubated with primary antibodies: lamin A/C antibody for mouse (#ab227176, Abcam), lamin A/C antibody for human (#MA3‐1000, Thermo Fisher Scientific), p16INK4a antibody for mouse (#ab211542, Abcam), p16INK4a antibody for human (#ab270058, Abcam), p21 antibody for mouse (#ab188224, Abcam), p21 antibody for human (#MA5‐31479, Thermo Fisher Scientific), p‐γH2AX antibody (#9718, Cell Signaling Technology), progerin antibody (#sc‐81,611, Santa Cruz) overnight at 4°C, followed by an anti‐rabbit or anti‐mouse IgG, HRP‐linked secondary antibody (Cell Signaling Technology). Blots were visualized using Pierce ECL Western Blotting Substrate (Thermo Fisher Scientific), and chemiluminescence was detected by a ChemiDoc Imaging System (Bio‐Rad). Protein expression was determined by densitometry using Image Lab (Bio‐Rad).

### 2.12 Quantitative real‐time PCR (qPCR)

Fibroblasts were washed with PBS and RNA was extracted using the miRNeasy Mini Kit (Qiagen), according to the manufacturer's protocol. cDNA was synthesized from 2 μg of total RNA using a RevertAid first‐strand cDNA synthesis kit (Thermo Fisher Scientific). qPCR primers were designed using Primer‐BLAST and are listed in Table S1. qPCR was performed, as previously described (Rogers et al., 2021), using SsoAdvanced Universal SYBR Green Supermix (Bio‐Rad) on a CFX Opus Real‐Time PCR System (Bio‐Rad).

### 2.13 Statistical analysis

Data are presented as mean ± standard error of the mean (SEM). Before conducting statistical analyses, the assumption of normality was assessed. Statistical significance was determined by a two‐way analysis of variance (ANOVA) or by an independent *t*‐test (where appropriate) using GraphPad Prism v10. Results with a *p* < 0.05 were considered statistically significant. Differences between groups were evaluated using a Bonferroni multiple comparison test.

## 3 RESULTS

### 3.1 Cardiac and skeletal muscle manifestations in humanized HGPS mice

HGPS mice exhibit significant differences in weight among males and females. In females, a decline in weight was observed beginning at 12 weeks of age, while in males, this decline was apparent at 16 weeks (Figure S1a). At 28 weeks of age, HGPS mice exhibited a substantial weight reduction of 30% in males and 25% in females compared to wild‐type littermates. Further investigation into tissue weights indicated a general reduction in various tissues in HGPS mice (Figure S1b). In males, the heart, TA, and spleen showed significant decreases in weight. Conversely, in females, only the spleen exhibited a significant weight reduction. When tissue weights were normalized by body weight, only the percentage of spleen weight relative to body weight was decreased in males (Figure S1c). Generally, spleen size is gradually reduced with aging. The reduction in spleen size is attributed to several factors, including a diminished metabolic rate, decreased immune function, and alterations in connective tissue, including fibrosis (Pantic et al., 2013; Suttie, 2006). Progerin, which induces tissue fibrosis, is likely responsible for the reduced size of the spleen, contributing to its shrinking and stiffening (Mu et al., 2020).

**FIGURE 1:**
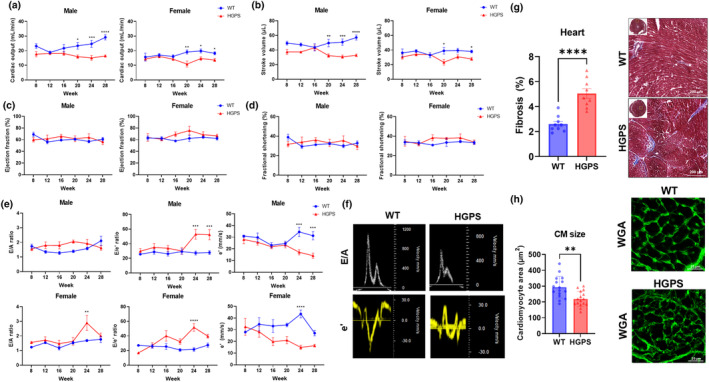
Analysis of heart function by systolic and diastolic echocardiography and fibrosis. (a) Cardiac output, (b) stroke volume, (c) ejection fraction, (d) fractional shortening, and (e, f) diastolic heart functions were measured every 4 weeks in wild‐type and HGPS mice. (g) Fibrosis was measured by Masson's trichrome staining (Scale bar = 200 μm). (h) Cardiomyocyte area was measured using hearts of 28‐week‐old wild‐type and HGPS mice by wheat germ agglutinin (WGA) staining (Scale bar = 25 μm). Mean ± SEM *p < 0.05, ** *p* < 0.01, ***p < 0.001, ****p < 0.0001. The results are the mean of the measurements (*n* ≥ 5).

Systolic and diastolic heart function were systematically assessed by echocardiography at 4 week intervals. In male HGPS mice, a decrease in cardiac output was observed beginning at 20 weeks of age, and in females, a significant difference in cardiac output was only noted at 24 weeks of age (Figure 1a). Similar to cardiac output, stroke volume of male HGPS was decreased beyond 20 weeks of age and in females at 20 and 28 weeks of age (Figure 1b). Typically, ejection fraction (EF) and fractional shortening (FS) decline with age, reflecting diminished pump function (Slotwiner et al., 1998). However, in G608G HGPS mice, no significant changes in EF and FS were observed, suggesting some processes associated with normal aging may not be active in HGPS mice (Figure 1c,d).

In addition to systolic heart function, we examined diastolic heart function in HGPS mice (Figure 1e,f). The E/A ratio, a key parameter reflecting early to late diastolic filling of the left ventricle, exhibited a notable increase only in female HGPS mice at 24 weeks of age, indicating impaired ventricular relaxation in this group. Furthermore, the E/e' ratio, the ratio of early diastolic mitral inflow velocity to early diastolic mitral annulus velocity, increased in male HGPS mice from 24 and 28 weeks of age, and at 24 weeks in females, suggesting greater myocardial stiffness and decreased ventricular relaxation. Additionally, the early diastolic mitral annular velocity, e', showed a consistent decrease in both males and females from 24 weeks of age. This reduction in e' represents impaired relaxation of the left ventricle during early diastole, contributing to compromised diastolic function in HGPS mice. These findings collectively show comprehensive cardiac dysfunction in HGPS mice, involving both systolic and diastolic parameters. The unique cardiac phenotype: decreased cardiac output and stroke volume with stable EF and FS, and concurrent changes in diastolic function highlights the complexity of the cardiovascular manifestations associated with progerin expression in HGPS.

In terms of structural alterations in HGPS hearts, echocardiography revealed alterations in diameter, volume, and left ventricular posterior wall thickness in diastole (Figure S2). Diameter and volume in diastole were reduced in both male and female HGPS mice at 28 weeks of age compared to wild‐type mice (Figure S2a,b). Additionally, left ventricular posterior wall thickness in diastole was increased in both male and female HGPS mice compared to wild‐type mice, suggesting myocardial hypertrophy (Figure S2c). The combination of decreased end‐diastolic diameter and volume, along with increased left ventricular posterior wall thickness in diastole, suggests geometric changes may, in part, underlie compromised function (Dorfman et al., 2007; Perhonen et al., 2001). We next analyzed cardiomyocyte cross‐sectional area in both wild‐type and HGPS mice at 28 weeks of age. In HGPS mouse hearts, cardiomyocyte cross‐sectional area was decreased compared to wild‐type mice (Figure 1h). Together, these data suggest abnormal cardiac geometry may underlie maladaptive changes in cardiomyocytes and altered function.

Masson's trichrome staining of hearts revealed increased cardiac fibrosis in HGPS mice (5.04%) compared to wild‐type mice (2.60%) (Figure 1g), which may contribute to increased myocardial stiffness. To verify myocardial collagen content and determine the relative contribution of collagen types I and III in the heart, hydroxyproline levels were measured, and collagen types I and III immunofluorescence were performed, respectively. Hydroxyproline content and expression of collagen types I and III were increased in the hearts of HGPS mice relative to wild‐type hearts (Figure S3a,b). Collagen type I was more abundant than type III, and may reflect a stiffer myocardium consistent with impaired relaxation.

**FIGURE 2:**
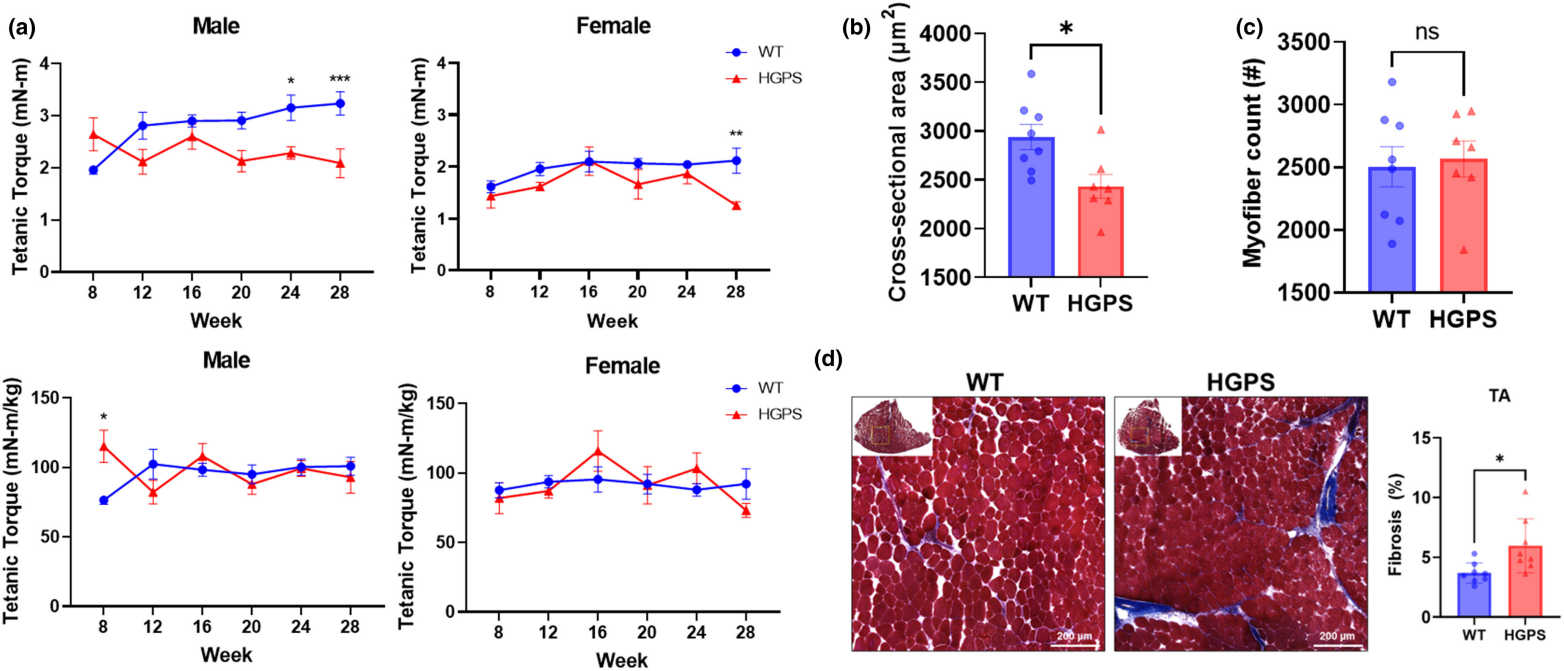
Muscle function and fibrosis of the tibialis anterior (TA) in wild‐type and HGPS mice. (a) Isomeric tetanic torque of the TA was measured every 4 weeks in wild‐type and HGPS mice. (b) Fibrosis of TA was measured by Masson's trichrome staining. Bar graphs showing (c) the myofiber cross‐sectional area and (d) myofiber count in TA of wild‐type and HGPS mice. Mean ± SEM *p < 0.05, **p < 0.01, ***p < 0.001, ****p < 0.0001. Scale bar = 200 μm.

To probe skeletal muscle function, isometric tetanic torque of the anterior crural muscles was measured in both HGPS and wild‐type mice every 4 weeks (Figure 2a). Absolute isometric tetanic torque decreased at 24 and 28 weeks of age in male mice and at 28 weeks in female mice. This effect was not observed when muscle torque was normalized to body weight, suggesting atrophy rather than contractile defects underlie decreased absolute torque production in HGPS mice. Indeed, histology revealed average myofiber cross‐sectional area was significantly reduced in HGPS mice compared to wild‐type mice (Figure 2b). No differences were observed in the total number of myofibers between genotypes, suggesting that the reduction in muscle torque was not due to a decrease in the overall number of muscle fibers but rather a consequence of changes in cross‐sectional area (Figure 2c). Masson's trichrome staining of TA muscles demonstrated an increase in fibrosis from 3.68% to 5.97% in HGPS mice and indicate structural abnormalities are also present in skeletal muscle of HGPS mice (Figure 2d). Furthermore, increased hydroxyproline content and upregulated expression of collagen types I and III were also observed in the TA of HGPS mice, relative to wild‐type mice (Figure S3a,c).

### 3.2 Phenotypic characterization of mouse and human HGPS fibroblasts

**FIGURE 3:**
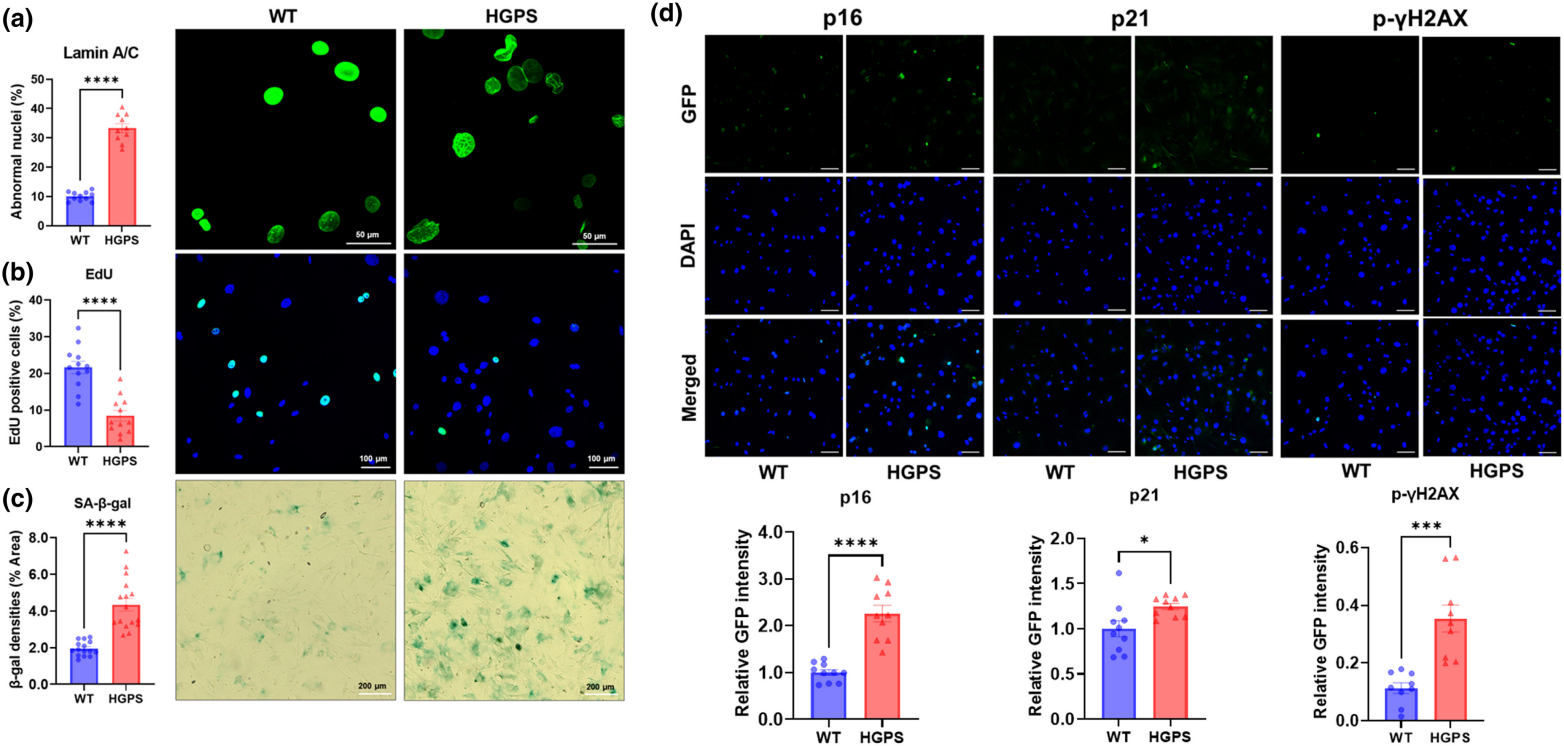
Characterization of fibroblasts from wild‐type and HGPS mice by staining of senescence‐associated markers. Mouse fibroblasts were stained for (a) lamin A/C (Green), (b) EdU (Green), (c) SA‐β‐galactosidase (Blue), and (d) DNA damage response markers (p16, p21, and p‐γH2AX with green). GFP intensity was normalized by cell number per field. Mean ± SEM *p < 0.05, **p < 0.01, ***p < 0.001, ****p < 0.0001. Scale bar = 100 μm.

Because the heart and skeletal muscle of HGPS mice were fibrotic, we questioned whether fibroblasts were altered. Generally, fibroblasts are responsible for producing and maintaining the extracellular matrix, which provides structural support to tissues. However, progerin expression leads to alterations in the composition and quality of the ECM associated with tissue stiffness and various connective tissue abnormalities (Mu et al., 2020). To characterize HGPS fibroblasts, we isolated primary fibroblasts from the skin of 28‐week‐old HGPS and wild‐type littermates. Lamin A/C staining revealed abnormally‐shaped nuclei in HGPS fibroblasts, a hallmark feature of progeric cells (Figure 3a). Further analysis revealed a decrease in cell proliferation, as measured by reduced EdU‐positive HGPS fibroblasts (Figure 3b). Additionally, the expression of SA‐β‐galactosidase was increased in HGPS fibroblasts (Figure 3c). We next sought to determine if these findings were reproduced in human HGPS fibroblasts. Indeed, similar to mouse fibroblasts, human HGPS fibroblasts exhibited abnormal nuclei with defective structure (Figure S4a), decreased EdU‐positivity and increased SA‐β‐galactosidase expression (Figure S4b,c). Thus, reduced cell proliferation and senescence are seen in both mouse and human HGPS fibroblasts.

Cellular oxidative stress induced by progerin trigger a DNA damage response (DDR) by stimulating p16 (CDKN2A), p21 (CDKN1A), and p‐γH2AX (Batista et al., 2023; Kubben & Misteli, 2017). The expression of p16, p21, and p‐γH2AX was elevated in mouse HGPS fibroblasts by immunocytochemistry (Figure 3d) and confirmed by Western blotting (Figure S5). Similar to mouse HGPS fibroblasts, human HGPS fibroblasts also express greater levels of p16, p21, and p‐γH2AX (Figures S4d and S6).

**FIGURE 4:**
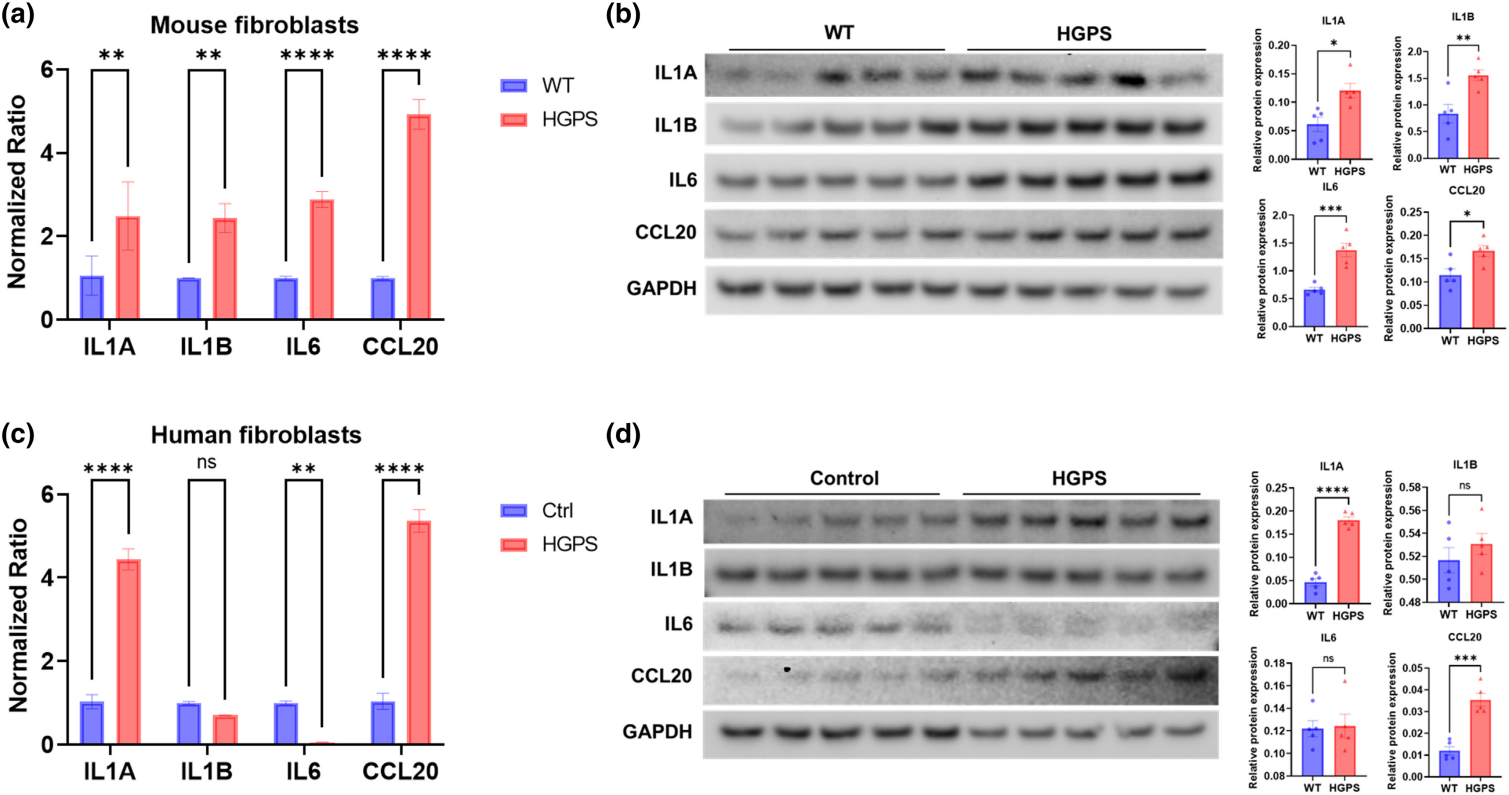
Analysis of senescence‐associated secretory phenotype (SASP) mRNA and protein expression in mouse and human fibroblasts. The expression of IL‐1α, IL‐1β, IL‐6, and CCL‐20 were measured by quantitative PCR and Western blotting in mouse fibroblasts (a and b) from wild‐type and HGPS mice and human fibroblasts (c and d) from control and HGPS. Mean ± SEM *p < 0.05, **p < 0.01, ***p < 0.001, ****p < 0.0001.

Senescent cells acquire a senescence‐associated secretory phenotype (SASP) characterized by the expression of pro‐inflammatory cytokines, growth factors, and proteases (Tchkonia et al., 2013). Therefore, we measured the relative mRNA and protein levels of SASP factors IL‐1α, IL‐1β, IL‐6, and CCL20 in both mouse and human fibroblasts (Figure 4). mRNA levels of IL‐1α, IL‐1β, IL‐6, and CCL20 were upregulated in mouse HGPS fibroblasts, 2.49‐, 2.44‐, 2.88‐, and 4.92‐fold respectively compared to wild‐type fibroblasts (Figure 4a). Furthermore, the protein expression of IL‐1α, IL‐1β, IL‐6, and CCL20 was significantly increased in mouse HGPS fibroblasts, relative to wild‐type fibroblasts (Figure 4b). In human HGPS fibroblasts, the expression of IL‐1α and CCL20 was increased by 4.44‐ and 5.36‐fold, respectively, compared to control fibroblasts (Figure 4c). In contrast, mRNA levels of IL‐6 was decreased by 94% in human HGPS fibroblasts compared to control fibroblasts (Figure 4c). The protein expression of IL‐1α and CCL20 showed increased expression in human HGPS fibroblasts, but no differences in IL‐1β and IL‐6 were detected between human HGPS fibroblasts and control fibroblasts (Figure 4d). Overall, the differential expression of SASP‐related genes observed between mouse and human HGPS fibroblasts indicate potential species‐specific variations in the inflammatory profiles associated with HGPS.

TGF‐β1 induces the differentiation of fibroblasts into myofibroblasts, which plays a crucial role in the production of collagen and other components of the extracellular matrix (Biernacka et al., 2011). The excessive accumulation of collagen may result in fibrosis, impairing normal organ function. Upon incubation of mouse fibroblasts with recombinant TGF‐β1, the mRNA expression of collagen 1A1 (COL1A1) was increased in both wild‐type and HGPS. However, COL1A1 expression was greater in TGF‐β1‐activated HGPS fibroblasts compared to TGF‐β1‐activated wild‐type fibroblasts (Figure S7). This observation highlights the fibrogenic characteristic of HGPS fibroblasts, which may underlie increased fibrosis observed in the heart and TA of HGPS mice.

## 4 DISCUSSION

In this study, we investigated the cardiac and skeletal muscle manifestations of HGPS in the humanized G608G mouse model. Echocardiography revealed cardiac dysfunction in HGPS mice, including decreased cardiac output and stroke volume, and impaired relaxation of the left ventricle, which was associated with cardiac fibrosis. Additionally, HGPS mice exhibited reduced absolute isometric tetanic torque, which was associated with muscle atrophy and increased fibrosis in the TA. At the cellular level, HGPS fibroblasts showed nuclear abnormalities, reduced cell proliferation, increased senescence markers, and fibrogenic activity.

The premature death of HGPS patients is generally caused by CVD (Hanumanthappa et al., 2011; Merideth et al., 2008; Olive et al., 2010; Rivera‐Torres et al., 2016; Song et al., 2014; Stehbens et al., 1999; Stehbens et al., 2001). HGPS mice generated by knockout of the Zmpste24 gene (Zmpste24^−/−^), needed for post‐translational processing of LMNA, does not exhibit major cardiovascular defects, except coronary artery fibrosis (Rivera‐Torres et al., 2016). Another wellstudied HGPS mouse model, harboring the mouse equivalent of the human LMNA G608G mutation (G609G; LMNA^G609G^) express progerin and exhibit prolonged QRS intervals, loss of VSMCs, and arterial stiffening (Del Campo et al., 2019; Kim et al., 2018; von Kleeck et al., 2021). The Lmna^G609G/G609G^ mouse model also exhibits reduced ejection fraction, FS, and diastolic dysfunction (Chen et al., 2023; Murtada et al., 2023). The humanized G608G HGPS mouse model characterized here is known to develop vascular alterations including a loss of VSMCs and vessel calcification, but other cardiac manifestations have not yet been reported (Lian et al., 2023; Varga et al., 2006). Here, we report cardiac dysfunction associated with cardiac fibrosis, a hallmark feature of clinical HGPS, is reproduced in G608G HGPS mice.

Despite the observed reductions in cardiac output and stroke volume, no changes were noted in ejection fraction or FS in HGPS mice. Zmpsts24^−/−^ HGPS mice showed increased ejection fraction and FS when compared to wild‐type mice (Rivera‐Torres et al., 2016). In clinical reports, diastolic dysfunction was observed in HGPS patients, yet changes in ejection fraction and FS were not evident (Prakash et al., 2018). However, several other HGPS animal models showed reduced ejection fraction and FS (Chen et al., 2023; Dorado et al., 2019; Kang et al., 2023). On balance, G608G HGPS mice appear to model the human HGPS cardiac phenotype especially well.

We also observed a reduction in isomeric tetanic torque, as well as increased fibrosis and atrophy of TA in HGPS mice. Zmpste24^−/−^ mice exhibit skeletal muscle weakness, muscle atrophy, and fibrosis (Greising et al., 2012; Mu et al., 2020). Moreover, the G608G HGPS mouse model showed altered cortical bone structure, increased muscle stiffness, and cartilage abnormalities (Cubria et al., 2020). In addition, two other HGPS mouse models, LMNA deficient mouse and Lmna^L530P/L530P^ mouse, exhibit muscular dystrophy in skeletal and cardiac muscles (Mounkes et al., 2003; Sullivan et al., 1999). Because fibrosis restricts proper muscle contraction leading to reduced strength and increased stiffness (Mahdy, 2019), we propose that increased fibrosis and muscle atrophy in TA could contribute to reduced muscle torque.

Because the heart and skeletal muscle of G608G HGPS mice are fibrotic, we were motivated to study fibroblasts. The presence of progerin induces abnormal nuclear morphology, together with alteration of nuclear membrane proteins and chromatin structure (Batista et al., 2023). These structural changes contribute to genomic instability and incidence of DNA damage. Furthermore, HGPS cells showed impaired recruitment of DDR molecules (Batista et al., 2023; Liu et al., 2013; Manju et al., 2006). As SASP includes pro‐inflammatory cytokines and chemokines, these inflammatory mediators can induce the production of reactive oxygen species, leading to DNA damage (Cisneros et al., 2023). Consequently, DNA damage, induced by progerin, may increase expression of p‐γH2AX at sites of double‐strand DNA breaks. The activation of p16 and p21 contributes to cellular senescence by inhibiting cell proliferation (Santin et al., 2020). As reported here, these pathological sequelae are active in mouse and human HGPS fibroblasts and support their utility as a model to study related therapeutics in vitro.

In conclusion, our data show the G608G HGPS mouse model exhibits cardiac and skeletal muscle features of clinical HGPS. This is the only HGPS mouse model shown to exhibit clinically‐relevant cardiac and skeletal muscle dysfunction. Furthermore, we observed nuclear abnormalities, decreased cell proliferation, and increased expression of senescence markers in HGPS fibroblasts from both mouse and human. Together, the G608G HGPS mouse model may be useful for studying experimental therapeutics both in vivo and in vitro.

## AUTHOR CONTRIBUTIONS

YH and RGR designed the experiments. YH, AR, NM, JA, WL, MF, and AO performed the experiments. YH and RGR wrote the manuscript. All authors read and approved the final manuscript.

## FUNDING INFORMATION

General laboratory support was provided by NIH R01 HL167921 to RGR.

## CONFLICT OF INTEREST STATEMENT

The authors declare that they have no competing interests.

## Supporting information


Data S1.

## Data Availability

All data are available in the main text or the supplementary materials.
