# Supplementary material for: Cardiac and skeletal muscle manifestations in the G608G mouse model of Hutchinson‐Gilford progeria syndrome
Source: Aging Cell. 2024 Jul 3;23(10):e14259. doi: 10.1111/acel.14259 (PMC11464102; doi:10.1111/acel.14259)
Supplement: Supplementary file 1 — Data S1. [file ACEL-23-e14259-s001.pdf]

Table S1. Primer sequences for quantitative real-time polymerase chain reaction (qPCR)

Species	Gene	F/R	Primer sequences (5′–3′)	Accession No.
Mouse	IL1A	F	GTCGGGAGGAGACGACTCTAA	NM_010554.4
		R	GTTTCTGGCAACTCCTTCAGC	
	IL1B	F	TGCCACCTTTTGACAGTGATG	NM_008361.4
		R	TGATGTGCTGCTGCGAGATT	
	IL6	F	CCCCAATTTCCAATGCTCTCC	NM_031168.2
		R	CGCACTAGGTTTGCCGAGTA	
	CCL20	F	TGAGAATGGCCTGCGGTG	NM_016960.2
		R	TCGTAGTTGCTTGCTGCTTCTG	
	COL1A1	F	TTCAGGGAATGCCTGGTGAA	NM_007742.4
		R	ACCTTTGGGACCAGCATCA	
	GAPDH	F	AGGTCGGTGTGAACGGATTTG	NM_001289726.2
		R	GGGGTCGTTGATGGCAACA	
Human	IL1A	F	CATTGGCGTTTGAGTCAGCA	NM_000575.5
		R	CATGGAGTGGGCCATAGCTT	
	IL1B	F	CAGAAGTACCTGAGCTCGCC	NM_000576.3
		R	AGATTCGTAGCTGGATGCCG	
	IL6	F	TGCAATAACCACCCCTGACC	NM_000600.5
		R	GTGCCCATGCTACATTTGCC	
	CCL20	F	CGAATCAGAAGCAGCAAGCAA	NM_004591.3
		R	GATTTGCGCACACAGACAACT	
	GAPDH	F	TTGCCCTCAACGACCACTTT	NM_001256799.3
		R	TGGTCCAGGGGTCTTACTCC	

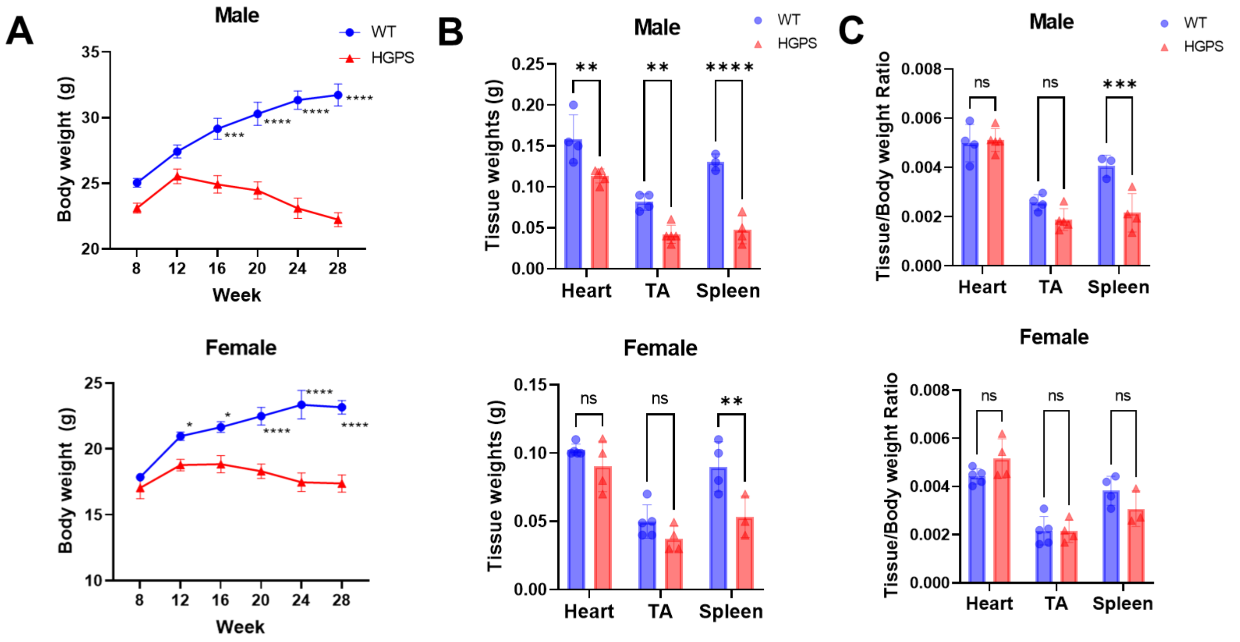

Figure S1. Body weight and tissue weights of wild-type and HGPS mice. (A) Growth curves for male and female wild-type and HGPS mice. Body weights were measured every 4 weeks up to 28 weeks. (B) Total tissue weights were measured at 28 weeks-old and (C) relative tissue weight to body weight was calculated. Mean ± SEM * $p<0.05$, ** $p<0.01$, *** $p<0.001$, **** $p<0.0001$.

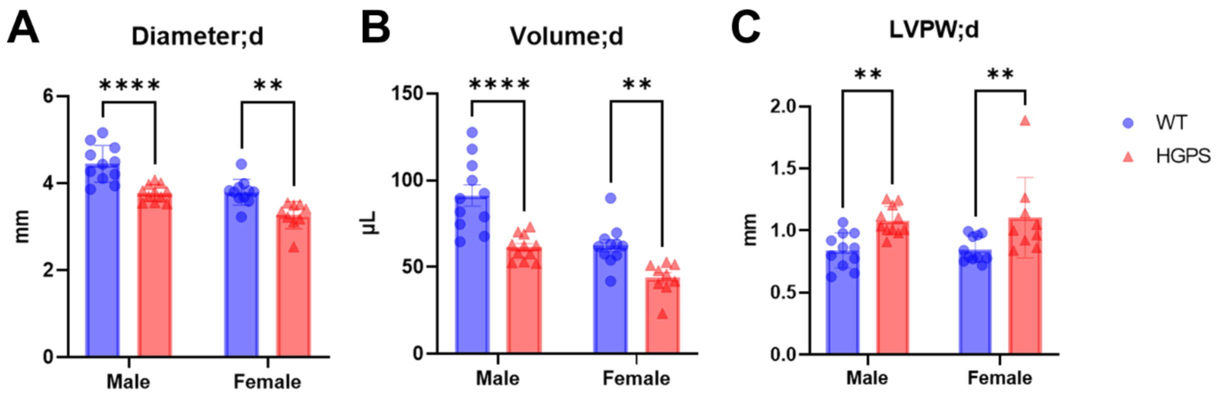

Figure S2. Echocardiographic properties in 28-week age of wild-type and HGPS mice. Heart geometric measurements include (A) diameter in diastole, (B) volume in diastole, and (C) left ventricular posterior wall thickness in diastole. Mean ± SEM * $p<0.05$, ** $p<0.01$, *** $p<0.001$, **** $p<0.0001$.

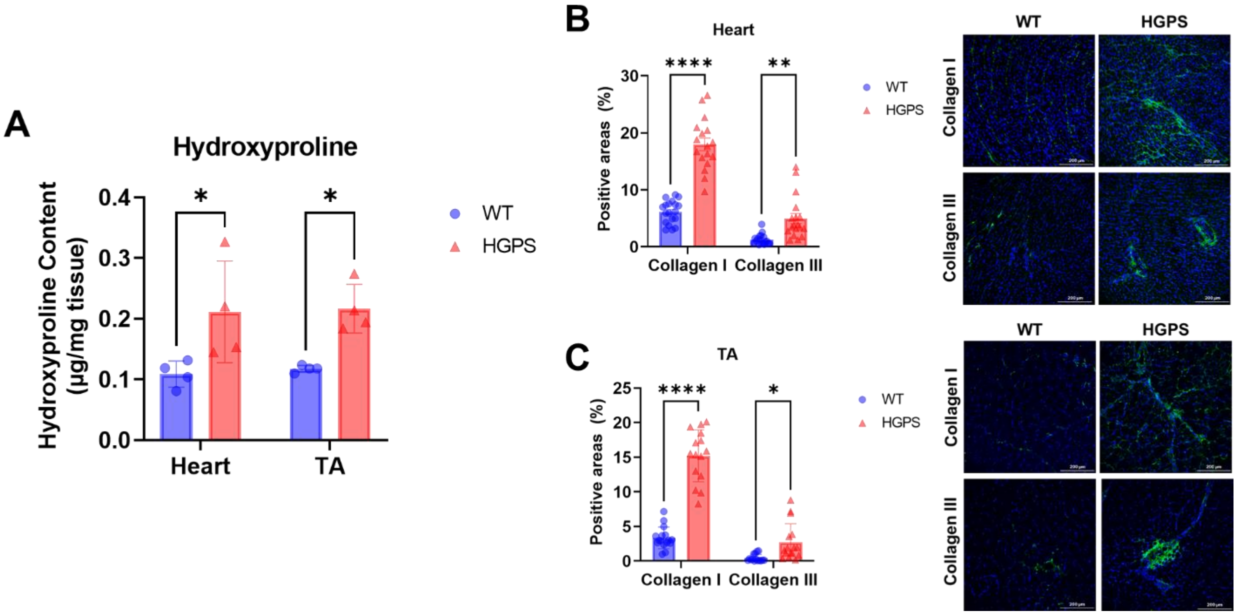

Figure S3. Collagen measurement in the heart and TA of wild-type and HGPS mice. (A) Hydroxyproline content was measured in the hearts and TA of 28-week-old wild-type and HGPS mice. Immunohistochemistry staining of collagen type I and collagen type III in (B) heart and (C) TA of wild-type and HGPS mice. Green fluorescence represents collagen type I and III and blue fluorescence represents the nucleus. Mean ± SEM * $p<0.05$, ** $p<0.01$, *** $p<0.001$, **** $p<0.0001$. Scale bar = 200 µm

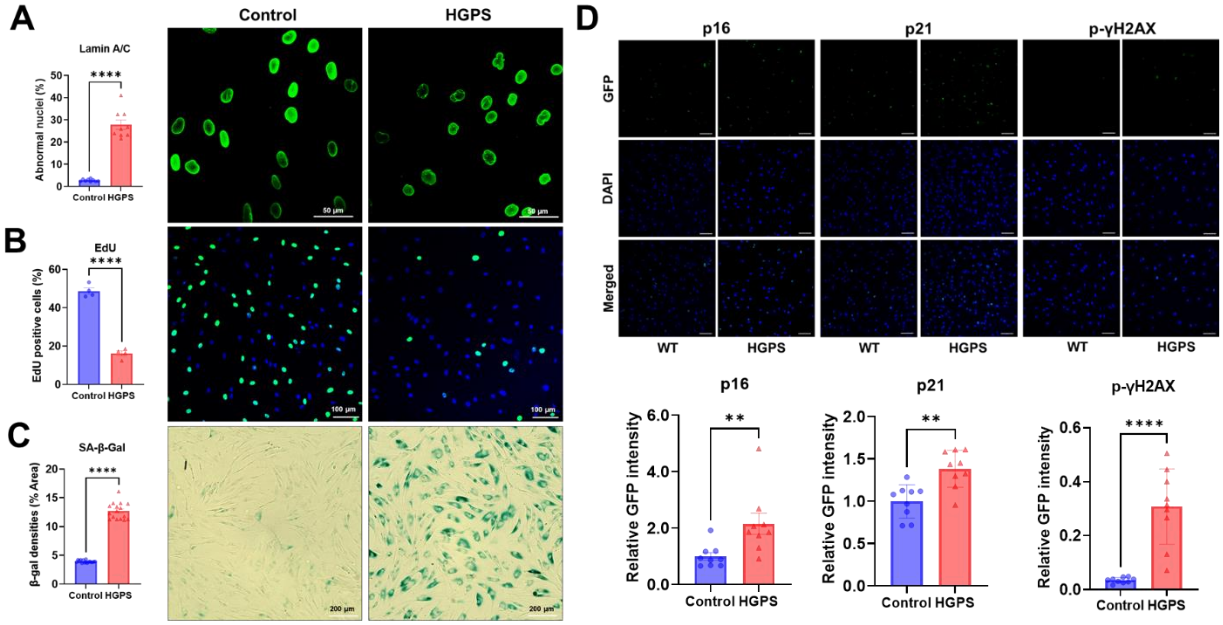

Figure S4. Characterization of fibroblasts from human control and HGPS donors by staining of senescence-associated markers. Human fibroblasts were stained for (A) SA-β-galactosidase, (B) Lamin A/C, (C) EdU, and (D) DNA damage response markers (p16, p21, p-γH2AX). The GFP intensity was normalized by cell number per field. Mean ± SEM * $p<0.05$, ** $p<0.01$, *** $p<0.001$, **** $p<0.0001$. Scale bar = 100µm

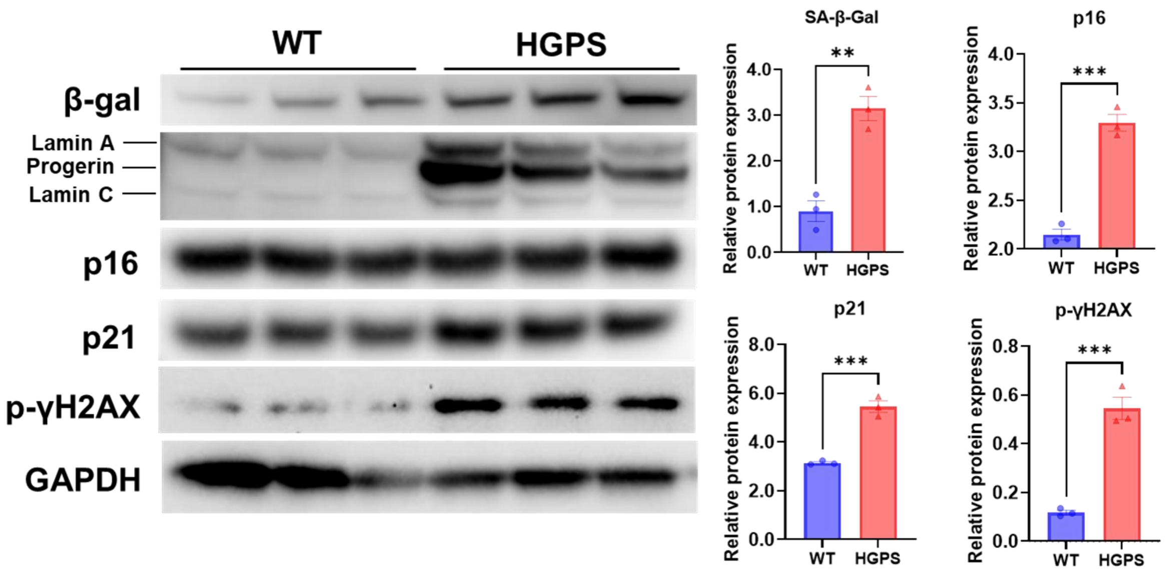

Figure S5. Western blotting analysis about senescence-associated markers in mouse fibroblasts from wild-type and HGPS mice. Total cell protein from mouse fibroblasts were analyzed by Western blotting using antibodies against β-galactosidase, Lamin A/C, p16, p21, p-γH2AX, and GAPDH. Mean ± SEM * $p<0.05$, ** $p<0.01$, *** $p<0.001$, **** $p<0.0001$.

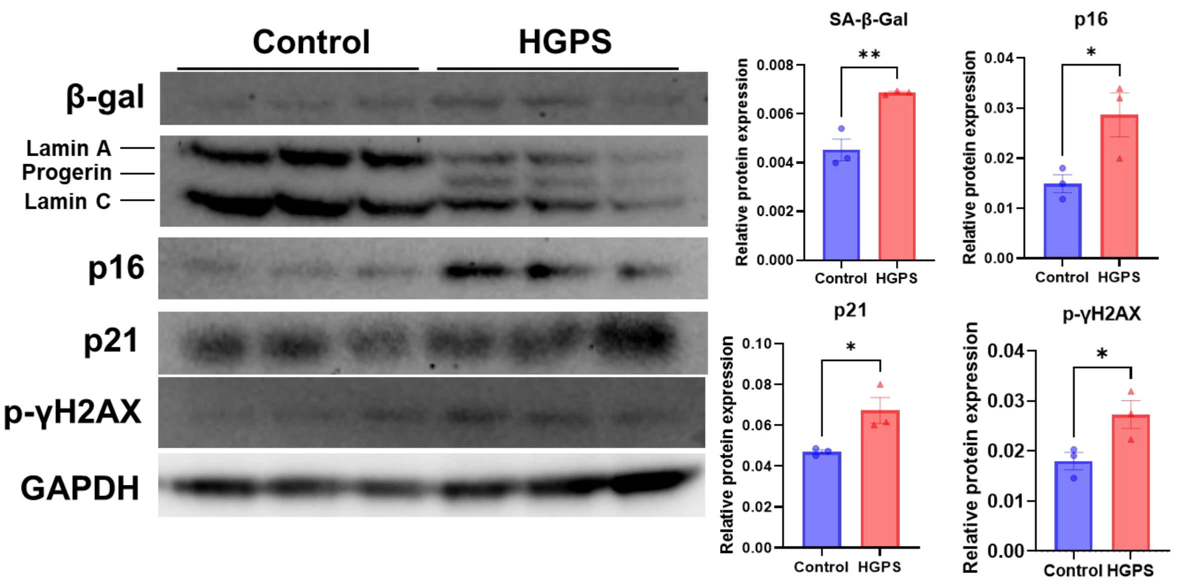

Figure S6. Western blotting analysis of senescence-associated markers in human fibroblasts from control and HGPS donors. Total cell protein from human fibroblasts were analyzed by Western blotting using antibodies against β-galactosidase, Lamin A/C, p16, p21, p-γH2AX, and GAPDH. Mean ± SEM * $p<0.05$, ** $p<0.01$, *** $p<0.001$, **** $p<0.0001$.

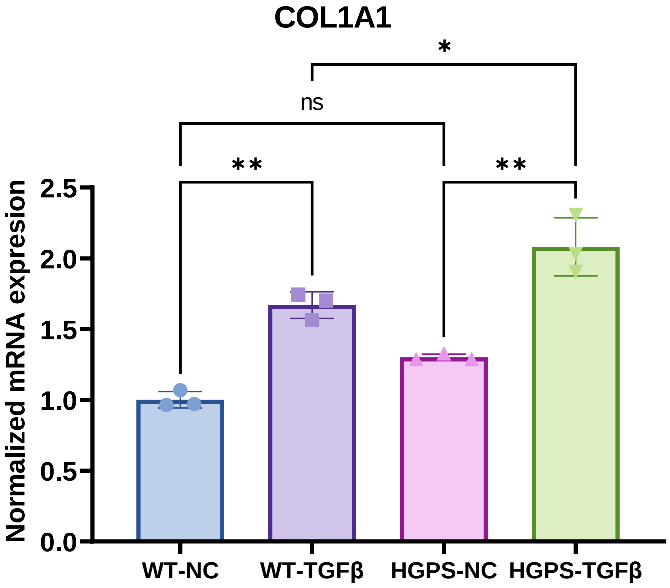

Figure S7. mRNA expression of COL1A1 in the mouse fibroblasts from wild-type and HGPS mice. Mouse fibroblasts were stimulated by NC (PBS) or 10 ng/mL of mouse recombinant TGF-β for 24 hours and the expression of COL1A1 was measured by qPCR. Mean ± SEM * $p<0.05$, ** $p<0.01$, *** $p<0.001$, **** $p<0.0001$.
